# Influence of Acrylic Acid and Tert-Dodecyl Mercaptan in the Adhesive Performance of Water-Based Acrylic Pressure-Sensitive Adhesives

**DOI:** 10.3390/polym12122879

**Published:** 2020-11-30

**Authors:** Irene Márquez, Núria Paredes, Felipe Alarcia, José Ignacio Velasco

**Affiliations:** 1 Lubrizol Advanced Materials, Applications Department, Camí de Can Calders, 13, 08173 Sant Cugat del Vallès, Spain; irene.marquez@lubrizol.com (I.M.); nuria.paredes@lubrizol.com (N.P.); 2 Department of Materials Science and Engineering, Universitat Politècnica de Catalunya (UPC BarcelonaTech), Poly2 Group, ESEIAAT, Carrer de Colom, 11, 08222 Terrassa, Spain; felipe.alarcia@hotmail.com

**Keywords:** acrylic PSA, emulsion polymerization, adhesion properties

## Abstract

Currently, pressure-sensitive adhesives (PSA) are used in more than 80% of all labels in the market today. They do not require any heat, solvent, or water to activate: It only takes light pressure to apply them to a product surface. Many products that come in glass bottles need labels that have staying power in harsh conditions. For that reason, it is necessary to have a good balance between all the polymer adhesive properties. In this study is described how adhesive properties of water-based PSA were affected by varying the amount of functional monomer acrylic acid (AA) and chain transfer agent, tert-dodecyl mercaptan (TDM). Four series of PSA were prepared by emulsion polymerization. Within each polymer series, the AA monomer proportion was held constant between 0.5 and 3.0 phm, and the fraction of the chain transfer agent was varied 0.0 to 0.2 phm. The results showed that the gel content decreased with the increase of the chain transfer agent and with the reduction of AA. All adhesives properties (tack, peel, and shear resistance) improved with increasement of the AA monomer. The increase of chain transfer agent caused decrease of the gel content resulting in higher peel resistance and tack values, but lower shear resistance values.

## 1. Introduction

In a short time, pressure-sensitive adhesives (PSA) have shown great growth in the label market [1]. This type of adhesives are characterized by their ability to adhere strongly to a wide variety of substrates at room temperature with the application of slight pressure in a short period of time [2]. One of its key advantages over other types of adhesives is that PSA allows for labels to be made and stored in reels, which improve the labelling process. Many products that come in glass bottles need labels that have staying power in harsh conditions. For this, it is necessary to have a good balance between the three adhesive properties most demanded by companies in this sector: Tack, peel, and shear resistance, mainly the last ones. Having both high peel resistance, good adhesion, along with high shear resistance and good cohesion, is challenging for the companies since they are opposite characteristics to each other. Normally, with an increase in adhesion comes a decrease in cohesion, and vice versa [3,4].

Amongst the PSAs, the acrylic systems are some of the most widely used in the label market. They offer much higher performance than natural and synthetic rubber adhesives like a higher transparency, temperature resistance, resistance to solvent and plasticizers, higher molecular weight and lower glass transition temperature (*T*_g_). This type of polymer is produced by emulsion polymerization, in addition to offering environmental safety due to the use of water as a solvent, is characterized by high polymerization rates compared to bulk or solution polymerizations [5,6]. The main commercial acrylic polymers are based in a low *T*_g_ monomer, (e.g., n-butyl acrylate (n-BA)), combined with a high *T*_g_ monomer (e.g., acrylic acid (AA)) or other functional monomer, like acrylonitrile (ACN) [7].

The level of adhesive strength is the result of a combination of interfacial and bulk properties. Obtaining good results in tack and peel resistance is convenient using polymers with polar functionality. On the other hand, to obtain good results in a shear test, it is necessary to obtain a high molecular weight [8]. According the bibliography, the presence of AA contributes to enhance all adhesive properties (peel, tack, and shear) since this has tendency to form networks through hydrogen bonding [9,10,11]. In a recent work, Gower and Shanks [12] showed that with the increase of AA, the molecular weight (*M*_w_) of the PSA increased, improving the cohesion and resulting in higher shear properties. Additionally, with the increase of AA, they obtained higher results in the peel resistance and tack values. In other work, Aubrey and Ginosatis [13] showed that the presence of 10 wt % of AA increased the interfacial adhesion of the acrylic PSA to glass by approximately 150%. Chan and Howard [14], they showed that with the increase of AA the tack values rose and that with 3–4 mol% of AA they obtained a maximum in tack values. Another advantage of using AA as a comonomer is that it provides colloidal stability to the polymer particle due to steric and electrostatic repulsion [15].

According the bibliography, another for to obtain a balance between peel and shear resistance may be by the incorporation of chain transfer agent, like tert-dodecyl mercaptan (TDM) [3]. This can control the molecular weight and the gel content formation. As a general rule, PSA with low molecular weights generate high tack, while middle range molecular weights influence on the peel resistance and high molecular weights affect the shear resistance. Mercaptans are the most common type of chain transfer agents, as well as the most efficient [16]. These promote polymer chain terminations and formation of new chains, thus resulting in low molecular weights. According the bibliography, this effect is called the “patching effect” [17,18]. Plessis and Arzamendi [19] showed that by increasing the concentration of the chain transfer agent from 0.00 to 0.15% the gel content decreased from 55 to 0%, as did the average molecular weight. This was reflected in the adhesive properties in such way that samples with 0% of gel showed excellent tack values and the samples with 32% of gel content showed higher peel and shear resistance values. However, samples with the highest gel contents showed reduced resistance to peeling and shear, as a consequence of lower molecular mobility. The same effect was also demonstrated by Gower and Shanks [20]. As the concentration of the chain transfer agent increased, the tack increased, and the shear resistance decreased due to the decrease in *M*_w_. Furthermore, samples that did not contain chain transfer agent did not show significant differences despite of they were having different composition. However, when the chain transfer agent was present, there was a strong dependence on the composition of the copolymer.

The general aim was developing PSA for glass bottle labels that meet the requirements currently demanded by the market. For that, a balance must be found between peel resistance, tack and shear resistance. The present study is a contribution of how adhesive properties of water based PSA can be affected by varying the amount of AA and TDM in order to find a good balance. Four series of PSA were prepared by emulsion polymerization to find an optimal balance in the adhesive properties, between tack, peel resistance, and shear resistance. For this purpose, the AA and TDM concentrations were varied, keeping the proportion of n-BA and ACN constant, and the adhesive properties as well as the gel content were investigated on the prepared polymers.

## 2. Materials and Methods

AA and n-BA provided by BASF (Ludwigshafen, Germany) as well as ACN provided by IMCD Benelux B.V. (Amsterdam, Netherlands), were used as comonomers in the polymerization. Tert-dodecyl mercaptan (TDM) provided by Chevron Phillips Company LP (Tessenderlo, Belgium) was used as a chain transfer agent. The anionic polymerizable emulsifier, Maxemul^TM^ 6112, based in a modified alcohol ether phosphate, provided by Croda (Mill Hall, PA, USA), was also used in the polymerization. Ammonium carbonate ((NH_4_)_2_CO_3_) provided by BASF (Ludwigshafen, Germany) was used as a buffer and ammonium peroxide sulfate ((NH_4_)_2_S_2_O_8_) supplied by United Initiators (Pullach, Germany) was used as a thermal initiator. A combination of tert-butyl hydroperoxide (TBHP), provided by Pergan (Bocholt, Germany), and sodium formaldehyde sulfoxylate (Bruggolite^®^ E01), from Brüggemann KG (Heilbronn, Germany), were used as a redox system to reduce free monomer at the end of the polymerization. A 12.5% ammonia solution, provided by Barcelonesa drugs and chemicals (Cornellà del Llobregat, Spain), was used to neutralize the adhesives. THF 99%, provided by Merck (Hohenbrunn, Germany), was used as a solvent.

For adhesion tests a polyethylene terephthalate (PET) of 12 µm provided by Polinas (Manisa, Turkey) with corona treatment as a surface activation treatment and Tintoretto qesso ultraWS^TM^ paper provided by Arconvert (Sant Gregori, Spain) were used as substrates to perform the tests.

### 2.1. Emulsion Polymerization

**Figure 1:**
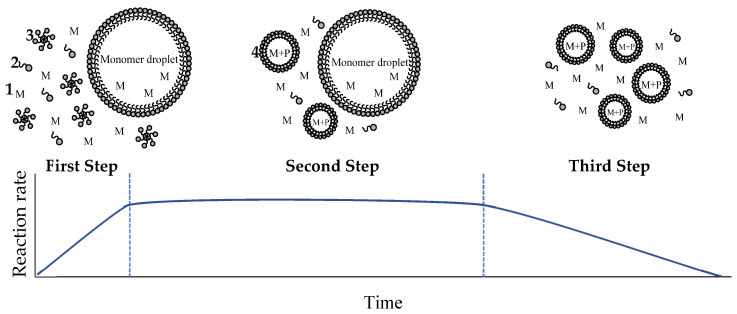
Schematic representation of the emulsion polymerization process and variation of reaction rate: (**1**) Monomer in aqueous phase; (**2**) free emulsifier; (**3**) micelles; and (**4**) polymeric particles.

Emulsion polymerization is a type of free-radical polymerization in a heterogeneous reaction mixture. Monomers, emulsifier, initiator and water are the main components of the mixture but also can be used buffers or chain transfer agents. Water acts as a continuous phase allowing the diffusion of species by the system. The process typically starts when the concentration of emulsifier reaches above its critical micelle concentration (CMC), forming micelles [21]. The initiator enters into the micelle where takes place the free radical propagation. The polymerization occurs inside micelle, it grows by monomer addition from monomer droplets outside and the polymer are formed. Emulsion polymerization carried out through three main steps as shown in Figure 1.

First step shows the particle formation. Radicals are generated from initiators and react with the monomers in aqueous phase forming small oligomers. These oligomers enter in the micelles forming the polymer particles. This phenomenon is called micellar nucleation. During this step, the particles number and the polymerization rate increase with time.

Second step starts when the micelles disappear, and the polymerization occurs in the polymer particles. The monomer droplets provide the monomers to the polymer particles where takes place the reaction. In this step, the polymer particles and the polymerization rate remain constant.

Finally, in the third step start when the monomer droplets disappear, and the monomers absorbed in the polymer particle polymerize. In this step the polymer particles remain constant, but the polymerization rate decrease as monomer concentration is reduced [22,23].

In this study, all polymers were prepared at 55% of solid content, adjusting the amount of water to keep this rate constant. The polymerizations were carried out by a semi-continuous process in a 2.5 L glass reactor at 82 °C with mechanical stirring at 100 rpm. The initial charge in the reactor consisted of 0.3 parts of (NH_4_)_2_CO_3_ per 100 parts by weight of monomer (i.e., 0.3 phm), 0.1 phm of emulsifier and a half of the total water. After heating and purging the reactor with N_2_, 0.5 phm of the initiator agent was introduced followed by a pre-emulsion composed by the monomeric system (Table 1), 1.2 phm of emulsifier and the remaining water. The pre-emulsion was added at a constant rate in 3 h

Once the pre-emulsion feed was completed, 0.1 phm of the initiator agent was added. One hour later, the same quantity of initiator was added again to help the polymerization process. Finally, 2 h later, the reactor was cooled down to 57 °C and a redox system was added TBHP/*Bruggolite^®^ E01* (0.2 phm/0.3 phm). Post-polymerization was allowed to take place during 4 h. Gas chromatography analysis indicated that the free monomer concentration was lower than 700 ppm.

### 2.2. Latex Characterization

The synthetized polymers were filtered through a 150 µm metallic filter and then analyzed to determine their physico-chemical characteristics.

The *T*_g_ values were experimentally determined by differential scanning calorimetry (DSC) using a DSC 1 STARe apparatus calibrated with an Indium standard. Samples of about 20 mg were initially placed in the crucibles and dried in an oven at 60 °C for 24 h to obtain dry test samples of about 10 mg. These samples were firstly heated at a rate of 20 °C/min from 25 °C to 200 °C, then held for 15 min at 200 °C and cooled to −65 °C at 20 °C/min. After stabilization for 15 min at −65 °C, the second heating was carried out at 20 °C/min up to 200 °C. The *T*_g_ value of each polymer was determined from the second heating curve as the intersection of the curve with the bisector of the baselines of the glassy and rubbery zones by the STAR method.

The gel content was defined as the polymer fraction insoluble in THF at 70 °C. To obtain this polymer fraction, it is necessary to form macromolecules with molecular weight higher than 7 × 10^6^ g/mol, according to the literature [24]. It was measuring by Soxhlet extraction for 24 h. This fraction was dried in an oven at 60 °C for 24 h to determine the gel content by using Equation (1), where W_1_ represents the initial weight of the filter, W_2_ the weight of the filter with the dry polymer and W_3_ the final dry weight of the filter after extraction [25].

(1) $$\mathrm{Gel}\;\mathrm{content}\;(\%)\;=\;\frac{W_{3}\;{-\;W}_{1}}{W_{2}{\;-\;W}_{1}}\;\times \;100$$

### 2.3. Adhesion Tests

The adhesive properties were evaluated through shear, peel and tack test. Using a motorized laboratory coater, RK K Control Coater provided by Lumaquin S.A. (Montornès del Vallès, Spain), equipped with a bar of 50 µm, 50 g/m^2^ of polymer was applied onto the substrates, which were subsequently dried in the oven for 1 min at 100 °C leaving a layer of polymer of approximately 25 g/m^2^. Standard sized tapes were cut for each type of test.

The peel resistance, defined as the force required to remove a tape from a test panel, was evaluated by means of the 180° peel test after 20 min and 24 h from the tape application. Tapes of PET and paper of 275 × 25 mm^2^ were applied onto glass panels. A Zwick/Roell Z 2.5 tensioner (Zwick Ibérica Equipos de Ensayos, S.L., Sant Cugat, Spain) was used at a constant speed of 300 mm/min. The average force to remove the tape and the failure mode were recorded [26].

Tack is the capacity of the adhesive to form bonds with a substrate with a brief contact under slight pressure. Tack was determined by the loop tack test with an AT1000 tensile tester equipment. A loop was formed with a PET and paper tape of 175 × 25 mm^2^ and held with the upper clamp. A controlled contact was made at a constant speed of 300 mm/min onto glass panels. The maximum force required to peel off the tape from the panel and the failure mode were recorded [27,28].

The shear resistance is defined as the capacity of the PSA tape to remain adhered under constant load applied parallel to the surface of the tape and substrate. This test consists in apply a standard area of PET and paper tape of 25 × 25 mm^2^ on a panel of stainless steel to 2° from the vertical and holding 1 kg until failure. The average time the tapes take to shear from the test panel were recorded [29].

Dynamic shear tests were performed at 5 mm/min with a Zwick/Roell Z 2.5 machine (Zwick Ibérica Equipos de Ensayos, S.L., Sant Cugat, Spain) on PET tapes adhered on untreated steel panels at 25 °C with a contact area of 12.5 × 12.5 mm^2^. The tape was applied 20 min before the test by means of a rubber roller with a mass of 2 Kg [30]. The shear stress vs. strain curves were recorded and the elastic modulus (G), the maximum stress (τ_m_) values and the deformation energy until failure were determined. The shear modulus was determined as the initial slope of the curve with the linear correlation coefficient (r^2^), which in all cases was higher than 0.999.

## 3. Results and Discussion

### 3.1. Physico-Chemical Properties

**Figure 2:**
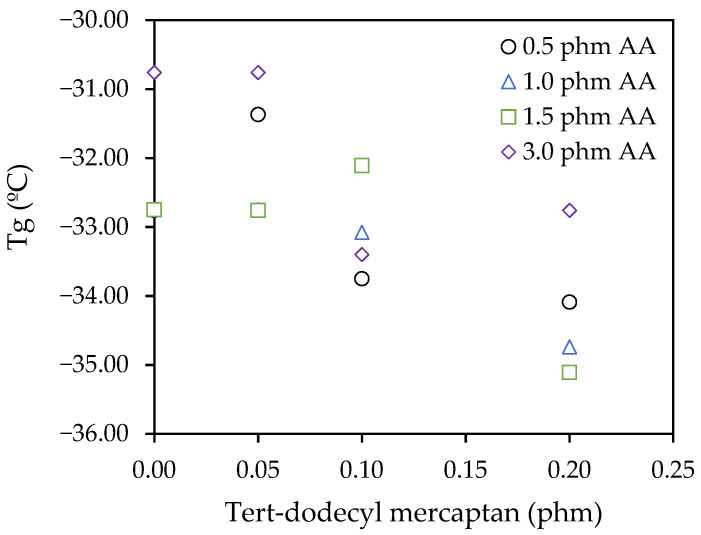
Effect of acrylic acid (AA) and TDM levels in the glass transition temperature (Tg).

The *T*_g_ results provided by DSC shown in Figure 2 shows a slight decrease in the *T*_g_ values when the amount of TDM was increased. On the other hand, changes in the amount of AA did not show a clear trend. However, these differences could not be considered significant since the proportions of AA and TDM varied were not significant compared to the rest of comonomers.

**Figure 3:**
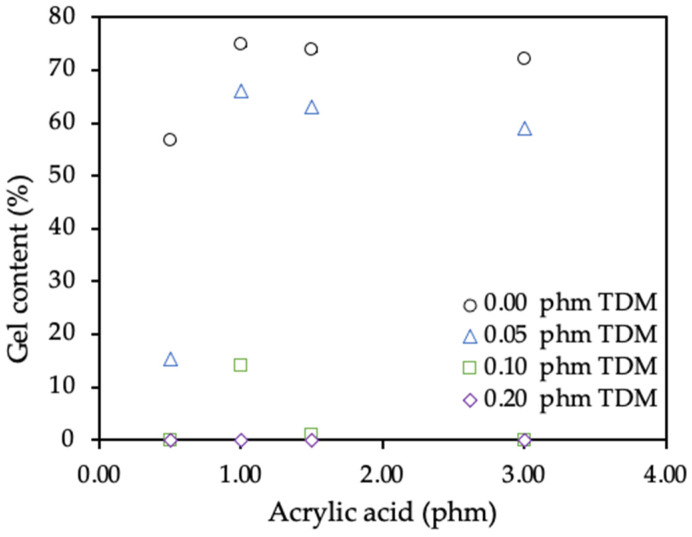
Effect of acrylic acid and tert-dodecyl mercaptan (TDM) levels in the gel content.

The gel content was studied by Soxhlet extraction and GPC. The results shown in the Figure 3 reflect the dependence of the gel content on the amount of copolymerized AA. The serie AA-1.0, with 1 phm of AA, showed the highest gel levels and the serie A, with 0.5 phm of AA, showed the lowest gel levels.

The samples where the TDM level was 0.00 phm showed the highest values of the gel content. As expected, the polymers with 0.2 phm showed the lowest gel content values. Nevertheless, within the same series where the amount of AA was kept constant, the TDM proportion most effective was using 0.20 phm and the less effective was using 0.00 phm. The proportions 0.1 and 0.2 phm of TDM were enough to avoid the gel fraction in almost all cases.

**Figure 4:**
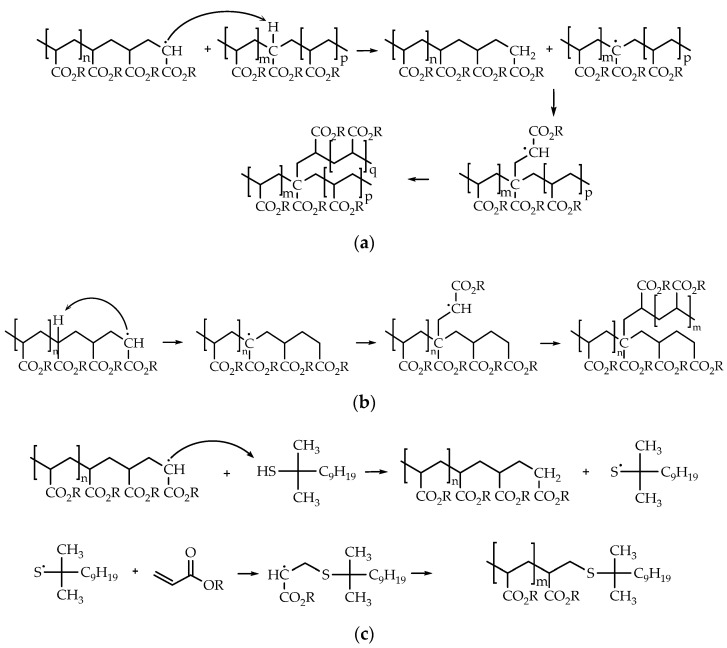
Mechanisms proposed for transfer to polymer reactions: (**a**) Intermolecular chain transfer to polymer. (**b**) Intramolecular chain transfer to polymer (backbiting). (**c**) Chain transfer to tert-dodecyl mercaptan.

Polymers containing AA form networks through hydrogen bonding. In previous studies, Cohen-Addad and Bogonuk determined that the gel content of AA-containing copolymers was due to the concentration of carboxylic acid groups [31]. However, more mechanisms are involved in the gel formation during emulsion polymerization. The acrylic monomers have two pathways to form branch points as shown Figure 4. The first pathway is by intermolecular chain transfer to polymer followed by termination by combination. In this the chain transfer reaction is between a polymer radical and a backbone polymer chain. The second pathway is a branching by intramolecular chain transfer (backbiting) [6]. This typical occurs by 6-membered ring transition state of a chain-end radical [32]. Both pathways generate tertiary radical species, but their propagation is slower than the secondary radicals located at the end of the polymer [33]. Although the intermolecular chain transfer is less prevalent than intramolecular chain transfer, it has higher effect. The intramolecular chain transfer generates short chain polymer branching and therefore its contribution is not significant [24,34,35,36]. On the other hand, the presence of TDM decreases the gel formation. It was due to the dominance of the chain transfer to TDM mechanism over the intermolecular chain transfer to polymer and termination by combination of molecules with long-chain branches as show the Figure 4c). These promote polymer chain terminations and formation of new chains, thus resulting in low molecular weights [37,38,39,40]. According to the bibliography, a growing macroradical abstracts a hydrogen atom from the chain transfer agent, giving a terminated polymer chain and a new radical is generated and is added to other monomer giving a new propagating species [41]. This way the chains are shorter and therefore the molecular weight and gel content are lower.

### 3.2. Adhesive Properties

Before their characterization, all polymers were adjusted to pH 7.5 by adding ammonia solution (12.5%) and the solid content to 50 wt. % with deionized water. The adhesive properties (peel resistance, tack, and shear resistance) were studied. Both AA and TDM had a strong influence on these properties.

**Figure 5:**
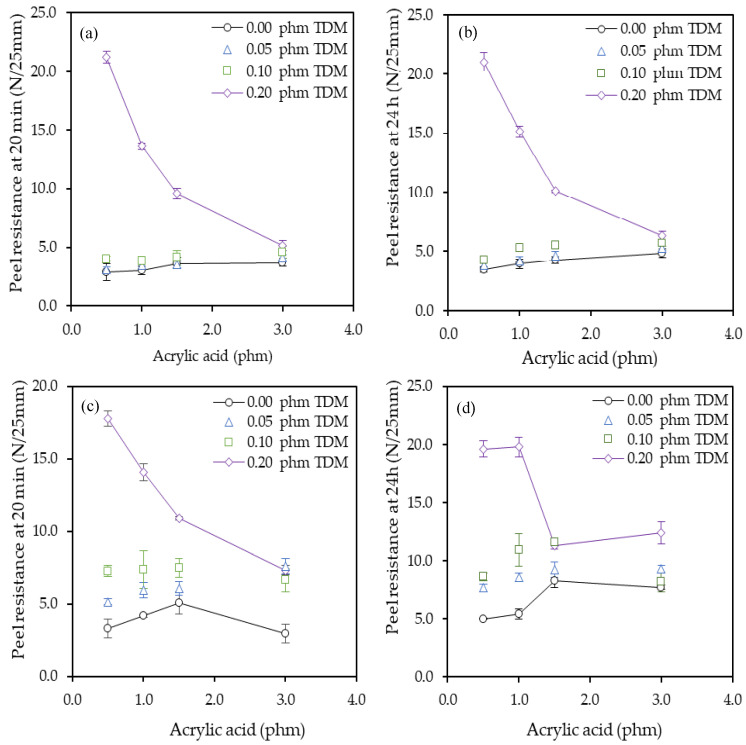
Experimental results of peel resistance at 20 min and 24 h (25 g/m^2^ of adhesive) on glass panels for (**a**,**b**) polyethylene terephthalate (PET), and for (**c**,**d**) paper tapes.

The effect of AA monomer and the TDM chain transfer agent on the peel resistance are shown in Figure 5. Both substrates used in this test, PET and paper, showed the same effect. The peel force values increased with the increase of AA and TDM. As expected, the values for the PET substrate were lower than the paper. As the paper is a porous substrate, the adhesive penetrates into the matrix paper resulting on a higher anchorage. As a result, the interfacial adhesive-substrate strength, i.e., adhesive strength was higher [42]. On the other hand, the deformation of the paper that takes place during the test is part of the fracture energy of the process. However, this does not happen with PET because it is an elastic substrate.

The values recorded after 24 h test were higher than the values after 20 min since over time the anchorage is better as shown in the Figure 5. The best balance between AA and TDM was using 0.5 phm of AA and 0.2 phm of TDM. This combination showed the highest peel values.

When the peel test was carried out on PET substrates onto glass, adhesive failures were obtained in all cases. However, when the test was carried out with paper, when the values were higher than 10 N/25 mm, cohesive failures were obtained, and for values lower, adhesive failures were obtained. This is most likely due because at low values the cohesive strength was higher than the adhesive strength and therefore occurs at the interface with the substrate. However, in the series of 3.0 phm of AA, transfer failures were observed in all cases. This could be due to the fact that with such high AA levels, the adhesive has more affinity for glass than for paper. The peel increased as the content of AA increased until up to a maximum with 1.5 phm AA since with 3.0 phm AA the values decreased. As expected, the adhesive material strength increases with increase of AA content and therefore the interfacial adhesion decreased. For this reason, in most cases, a maximum peel value was obtained 1.5 phm instead of with 3 phm [43]. On the other hand, the increase of TDM increased the peel values which was mainly attributed to the decrease of gel content. Since the increasing of amount of TDM decreased the chain length, improved their mobility and their interaction with the substrate [3]. However, with 0.2 phm of TDM, the best results were obtained with 0.5 phm AA. Unlike the rest of the proportions, with 0.2 phm of TDM the peel values decreased as the amount of AA increased.

Tack showed the same tendency as the peel resistance. Tack property is determined by the *T*_g_ and the molecular weight of the resulting polymer. Since in this case there were not significant variations in the *T*_g_ of the samples that were studied, the changes between the different samples only were due to changes in the composition.

**Figure 6:**
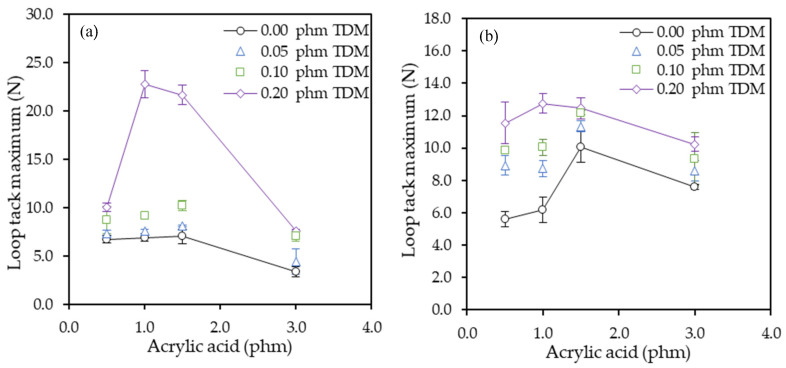
Effect of the acrylic acid and tert-dodecyl mercaptan (TDM) in the loop tack test on glass panels for (**a**) PET and (**b**) paper tapes.

Figure 6 illustrate the maximum values obtained for both substrates. Both substrates showed the same tendency. In general, the values for the PET substrate was lower than the paper. The anchorage in the paper was higher than in PET due to a higher proportion of adhesive penetrated in its porous and by doing that, adhesive strength was higher. However, the series with 0.20 phm of TDM showed higher values with PET substrate than with paper.

The tack increased as the content of AA increased until up to a maximum with 1.5 phm AA. The tack values of samples synthetized with 3.0 phm AA were similar to those of 1 phm AA. As seen in the previous section, the gel content, and therefore the cohesion strength, increased by increasing the AA proportion.

Tack is a property governed by low molecular weight fractions. If the gel content increases, the fraction of low molecular chains becomes lower producing a negative effect on tack. On the other hand, the presence of TDM considerably increased the tack values, as expected, due to the decrease in both the gel content and the *M*_w_ [44]. The best results were obtained with 0.2 phm of TDM, especially with polymers with 1.0 and 1.5 phm of AA.

**Figure 7:**
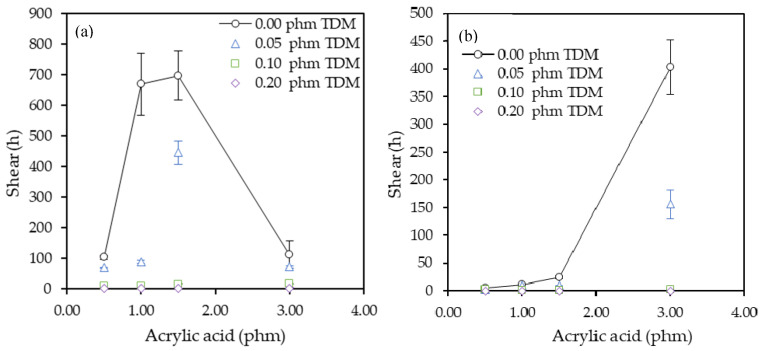
Effect of the acrylic acid and tert-dodecyl mercaptan (TDM) in the static shear test on steel panels for (**a**) PET and (**b**) paper tapes.

Figure 7 shows that static shear resistance of the polymers synthetized increased with AA level but decreased with the TDM level. Both substrates used in this test, PET and paper, showed the same effect. This test allows to determine the internal strength of the adhesive, i.e., the cohesive strength. As seen in the peel and tack results, the adhesive-substrate bond strength varies according to the substrate and the same occurs with the shear results. As expected, in this case, the PET substrate values were higher than the paper. As paper is a porous substrate, the anchorage interface between adhesive and substrate is higher than the internal strength of the adhesive because a higher proportion of the adhesive penetrates into the matrix paper. This makes the internal proportion of adhesive less and therefore the cohesive strength less too. However, the PET substrate has a more uniform surface which makes the adhesive-substrate bond lower but the internal strength higher.

With the increase of AA, the gel content and therefore the cohesive strength increased. The best results were obtained using 1.5 phm of AA without TDM for PET tapes and 3.0 phm of AA without TDM for paper tapes. When the TDM was added to the system, the gel content decreased as the proportion of chain transfer agent increased and this was reflected in the decrease in shear resistance. The samples with 0.20 phm of TDM showed the lowest gel content and probably the lowest *M*_w_. These ones showed the lowest shear resistance since their cohesive strength was poor.

**Figure 8:**
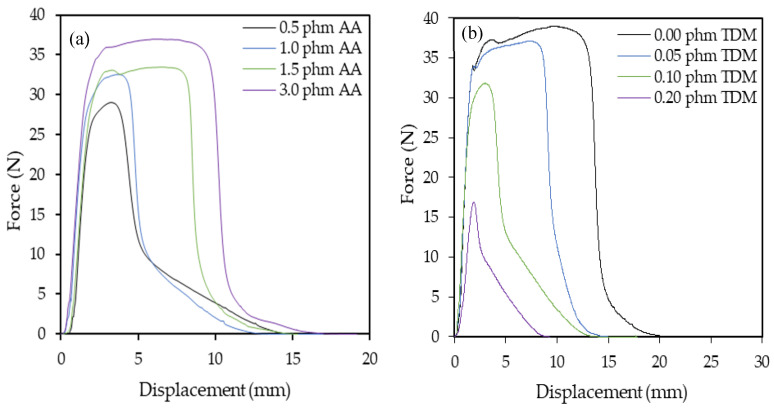
Experimental curves of dynamic shear test for (**a**) samples with constant tert-dodecyl mercaptan (TDM) ratio (0.05 phm) and (**b**) samples with acrylic acid (AA) ratio constant (3.0 phm).

The same effects were reflected in the dynamic shear resistance results, as shown in Figure 8.

Keeping the TDM ratio constant, with the increase of the proportion of AA, a higher force was necessary to remove the tape from the substrate test since the internal cohesion increase when the gel content increased. However, as was expected, keeping the AA ratio constant, with the increase of the proportion of TDM, a lower force was necessary to remove the tape from the substrate test since the cohesion decreasing when the gel content decrease due to the presence of the chain transfer agent.

**Figure 9:**
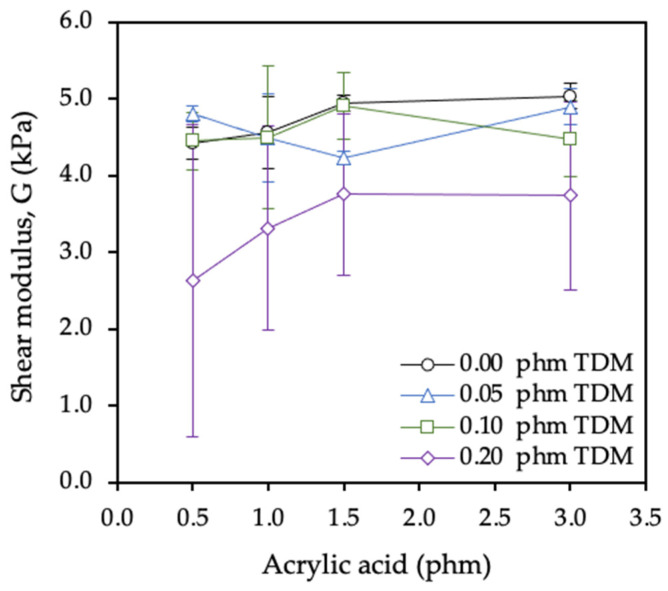
Effect of acrylic acid and tert-dodecyl mercaptan (TDM) contents on the shear modulus.

As shown in Figure 9, the shear elasticity modulus (G) did not significantly vary with the AA content. It is worth noting that the series with the highest TDM content (0.2 phm) revealed slightly lower G values as expected from its lower gel content.

**Figure 10:**
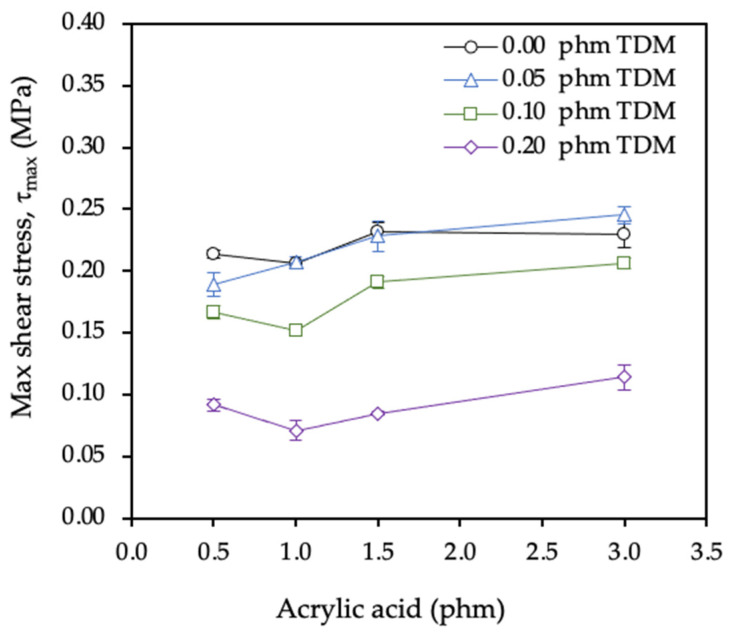
Effect of acrylic acid and tert-dodecyl mercaptan (TDM) on the shear strength of the adhesive.

The maximum shear stress recorded in the dynamic shear test (Figure 10) increased slightly with AA and decreased with the TDM content. This is in good agreement with the known function of the AA to increase the gel content and of the mercaptan to reduce the molecular weight through the chain transfer mechanism.

**Figure 11:**
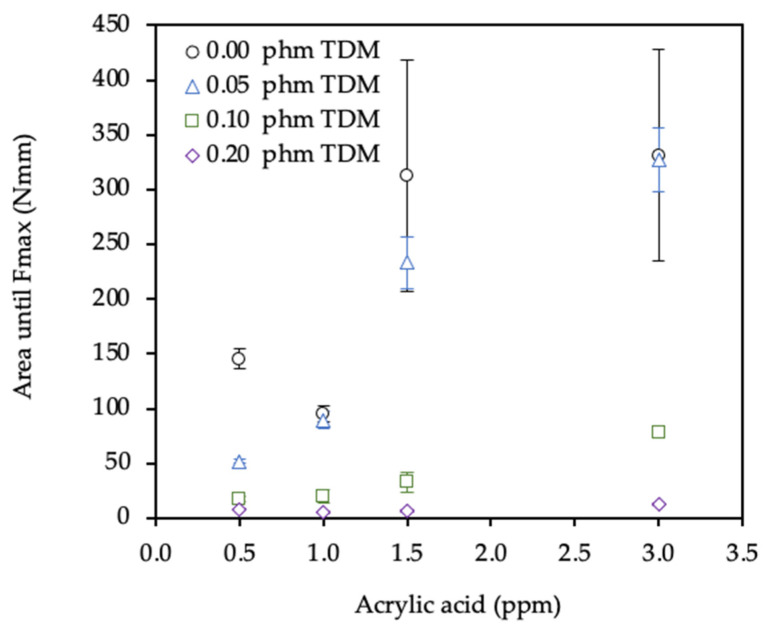
Effect of acrylic acid and tert-dodecyl mercaptan (TDM) on the deformation energy until failure of the adhesive.

Finally, the toughness of the adhesives was determined as the deformation energy up to the adhesive failure in the dynamic shear test and was calculated from the area under the curve force-displacement up to the maximum force value. As shown in Figure 11, it was required more energy in those samples synthesized without chain transfer agent (0.0 phm TDM) and with the maximum amount of AA (3.0 phm). In other words, those with the highest gel content had the highest cohesion and therefore the energy need to break the internal part of the adhesive had to be greater.

In general, the increase of the AA levels in these polymers increased all adhesive properties (peel resistance, tack, and shear resistance). However, the TDM made increase the adhesive strength and decrease the cohesive strength of the studied PSA. The presence of this compound generated chains with low molecular weight. This one improves the properties related to the interaction between the adhesive and the substrate (peel and tack). However, it decreased the intermolecular attractive forces within the adhesive, cohesive strength. Finally, the best balance for labels with both substrates between peel resistance, tack and shear resistance was using 1.5 phm of AA and 0.05 phm of TDM

## 4. Conclusions

To obtain the ideal balance between the three adhesive properties most demanded by the label market (peel resistance, tack and shear resistance), small amounts of AA and TDM were changing in a base formulation. Different acrylic PSA were prepared by emulsion polymerization. The results showed that the variation in *T*_g_ values were insignificants since the proportions of AA and TDM varied were not significant compared to the rest of comonomers. The gel content decreased with the increase of the chain transfer agent and with the reduction of AA. The proportions 0.1 and 0.2 phm of TDM were enough to avoid the gel fraction in almost all cases. It was due to the dominance of the chain transfer to TDM mechanism over the intermolecular chain transfer to polymer and termination by combination of molecules with long-chain branches. In general, all adhesive properties (peel resistance, tack and shear resistance) rose with the increase of proportion of AA monomer since this has tendency to form networks through hydrogen bonding. The peel force showed a maximum with 0.5 phm of AA and 0.20 phm of TDM with both substrates. The tack force increased four times with the combination of 1.0 phm of AA and 0.20 phm of TDM in both substrates. These combinations did not get gel content and probably, they would have a low Mw. With more AA and less TDM, the gel content was a bit much high and probable the Mw too, decreasing the adhesion of the polymer and increasing the cohesive strength. As expected, with 1.5 phm of AA and 0.00 phm of TDM, the maximum value of static shear was obtained for PET tapes and with 3.0 phm of AA and 0.00 phm of TDM, the maximum value of static shear was obtained for paper tapes.

The dynamic shear resistance test showed the same results that in the static shear test. As expected, the best results of G, maximum shear stress and deformation energy until failure were obtained with the highest AA level, 3.0 phm, and in absence of TDM. With 0.00 phm of TDM were obtained the highest gel content and therefore, the highest cohesion strength.

Finally, after the present work, an optimized PSA formulation based on the adhesion properties balance was found to be that synthetized with 1.5 phm AA + 0.05 phm TDM. With regard to the simpler PSA formulation usually used for glass bottle labels (0.5 phm AA), the new formulation increased the peel and tack forces 45% and 20% respectively on PET, and 85% and 100% respectively on paper substrate, as well as increased more than 4 and 24 times on PET and paper respectively the time to failure under static shear.

## Figures and Tables

**Table 1:** Monomer composition (phm): Acrylic acid (AA), n-butyl acrylate (n-BA), acrylonitrile (ACN), and tert-dodecyl mercaptan (TDM).

Series	AA	n-BA	ACN	TDM
AA-0.5	0.5	93.0	6.0	0.00
				0.05
				0.10
				0.20
AA-1.0	1.0	93.0	6.0	0.00
				0.05
				0.10
				0.20
AA-1.5	1.5	92.5	6.0	0.00
				0.05
				0.10
				0.20
AA-3.0	3.0	91.0	6.0	0.00
				0.05
				0.10
				0.20
